# The nature of relapse in schizophrenia

**DOI:** 10.1186/1471-244X-13-50

**Published:** 2013-02-08

**Authors:** Robin Emsley, Bonginkosi Chiliza, Laila Asmal, Brian H Harvey

**Affiliations:** 1 Department of Psychiatry, Faculty of Medicine and Health Sciences, Stellenbosch University, Tygerberg, Cape Town, 7505, South Africa; 2 Center of Excellence for Pharmaceutical Sciences, School of Pharmacy (Pharmacology), North West University, Potchefstroom, South Africa

**Keywords:** Schizophrenia, Relapse, Antipsychotics, Discontinuation, First-episode, Dopamine

## Abstract

**Background:**

Multiple relapses characterise the course of illness in most patients with schizophrenia, yet the nature of these episodes has not been extensively researched and clinicians may not always be aware of important implications.

**Methods:**

We critically review selected literature regarding the nature and underlying neurobiology of relapse.

**Results:**

Relapse rates are very high when treatment is discontinued, even after a single psychotic episode; a longer treatment period prior to discontinuation does not reduce the risk of relapse; many patients relapse soon after treatment reduction and discontinuation; transition from remission to relapse may be abrupt and with few or no early warning signs; once illness recurrence occurs symptoms rapidly return to levels similar to the initial psychotic episode; while most patients respond promptly to re-introduction of antipsychotic treatment after relapse, the response time is variable and notably, treatment failure appears to emerge in about 1 in 6 patients. These observations are consistent with contemporary thinking on the dopamine hypothesis, including the aberrant salience hypothesis.

**Conclusions:**

Given the difficulties in identifying those at risk of relapse, the ineffectiveness of rescue medications in preventing full-blown psychotic recurrence and the potentially serious consequences, adherence and other factors predisposing to relapse should be a major focus of attention in managing schizophrenia. The place of antipsychotic treatment discontinuation in clinical practice and in placebo-controlled clinical trials needs to be carefully reconsidered.

## Introduction

Schizophrenia is a chronic and disabling illness, with the majority of patients experiencing multiple relapses during the course of the illness [1]. Relapse, characterised by acute psychotic exacerbation, may have serious implications. For example, there is a risk of patients harming themselves or others, of jeopardising personal relationships, education or employment status [2], and of further stigmatisation of the illness. Additionally, relapse may carry a biological risk. It has been proposed that active psychosis reflects a period of disease progression insofar as patients may not return to their previous level of function and treatment refractoriness may emerge [3,4]. Multiple factors contribute to increasing the risk of relapse. In a systematic review and meta-analysis of longitudinal studies it was found that non-adherence with medication, persistent substance use, carers' criticism and poorer premorbid adjustment significantly increased the risk for relapse in first-episode psychosis [5]. In a prospective, 5 yr follow-up of first-episode psychosis patients it was found that the most common risk factor by far was antipsychotic medication discontinuation [1]. In clinical settings disengagement from treatment is common, even and perhaps particularly, in the early stages of illness [6-9]. In recognition of the associated risks, improving medication adherence and relapse prevention have been emphasised as key components of the management of schizophrenia [2]. However, there remain many unanswered questions regarding the nature of relapse making further study of this component of the illness imperative. During recent studies of relapse in schizophrenia that we conducted [10-12], we were struck by several observations:

1. Relapse rates are very high after treatment discontinuation, even after a single episode of psychosis.

2. A longer treatment period prior to discontinuation does not reduce the risk of relapse.

3. Many patients relapse very soon after treatment discontinuation.

4. The transition from remission to relapse may be abrupt, with few early warning signs.

5. Once illness recurrence occurs symptom severity rapidly returns to levels similar to the initial psychotic episode.

6. While most patients respond promptly to re-introduction of antipsychotic treatment after relapse, the response time is variable and frank treatment failure may emerge in a subset of patients.

In this article we critically examine each of these points and their clinical implications, and speculate on some underlying neurobiological mechanisms. We have not attempted to cover all of the important aspects such as risk-factors for, and predictors of relapse, and our focus is on relapse in the earlier phase of illness.

## Review

### Relapse rates after treatment discontinuation in first-episode schizophrenia

The rationale for clinicians to consider antipsychotic discontinuation after successful treatment of a first-episode of schizophrenia is based on three major considerations. First, there is a supposition that a substantial portion (perhaps 10–20%) of patients never experience a recurrence after a single psychotic episode [13], and will therefore not require ongoing maintenance treatment. Second, there is a considerable side-effect burden associated with antipsychotics. Prior to the availability of the second generation antipsychotics (SGAs) physicians were primarily concerned with the risk of motor disorders, particularly tardive dyskinesia (TD). In fact, it was recommended that antipsychotics should not be prescribed over a long period of time without adequate justification, for both clinical and medico-legal reasons [14]. Shortly after the introduction of the SGAs the hope was expressed that these agents, with their reduced propensity for motor side-effects, would carry a small enough risk to warrant indefinite maintenance treatment [15]. However, it is now recognized that these agents carry a significant side-effect burden of their own, including weight gain and its metabolic concomitants [16] and hyperprolactinaemia [17]. Also, it has been reported that rates of TD with SGAs, although lower than with conventional antipsychotics, are not as low as originally thought [18]. A third important reason for considering antipsychotic withdrawal is that clinicians may have difficulty in convincing patients that indefinite treatment is indicated after a single episode of psychosis, particularly those who have responded favourably to treatment. Treatment disengagement is common early in the illness, and the decision to discontinue medication is largely patient driven [19]. It could be argued that planned medication withdrawal with careful follow-up is preferable to treatment disengagement or covert non-adherence.

When determining the most appropriate maintenance treatment plan for an illness one aspect that needs to be considered is the risk of recurrence after discontinuation of treatment. While studies of treatment discontinuation consistently report higher relapse rates than with maintenance treatment, results differ widely. A comprehensive review of relapse after antipsychotic withdrawal in schizophrenia included 4365 patients from 66 studies published between 1958 and 1993 [20]. The authors found a mean cumulative relapse rate of 52% (ranging from 0% to 100%) over a mean follow-up period of 6.3 months (ranging from 0.5 to 24 months) for patients withdrawn from antipsychotics, versus 16% for those patients who continued to receive maintenance antipsychotic treatment. How-ever, these studies were conducted between 1958 and 1992, and interpretation of the results is hampered by inconsistencies in aspects such as diagnostic criteria, duration of treatment prior to antipsychotic discontinuation, degree of response at the time of discontinuation, criteria used to define relapse, and perhaps most significantly, the duration of follow-up periods. Furthermore, all but two of the 66 studies reported in the Gilbert et al. review [20] were conducted in chronic, multi-episode samples.

We performed a systematic review of the literature to identify studies that investigated relapse rates after treatment discontinuation after a first-episode of schizophrenia. We used the following key words: “first-episode schizophrenia or first-episode psychosis” combined with “discontinuation, intermittent treatment”. We also checked the reference lists of the relevant publications. We identified eight studies, details of which are summarised in Table 1. As with the multi-episode samples, these studies report varying rates of relapse, with four reporting symptom recurrence rates of close to 80% at 12 months after discontinuation [10,21-23] and two reporting rates of about 95% at 24 months [10,21]. Reasons for the lower reported recurrence rates in the other studies are not entirely apparent, although the Kane et al. study [24] had a high dropout rate (40%) and these patients were considered not to have relapsed; and in the Wunderink et al. study [25], of 65 patients randomized to the discontinuation arm 14 (21.5%) did not have symptom recurrence. Sixteen (24.6%) relapsed and 5 (7.7%) had mild symptom recurrence. However, in 30 subjects (46.2%) in the discontinuation arm antipsychotics were not discontinued due to physician concerns of worsening of psychosis. Taken together, these results suggest that even after a single episode of psychosis the vast majority of patients will experience symptom recurrence if followed up for a sufficient period of time.

**Table 1:** Studies reporting symptom recurrence rates after treatment reduction/discontinuation after a single episode of psychosis

**Authors**	**Sample size**	**Treatment duration**		**Symptom recurrence rates**				**Comparator recurrence rate**
			**9 months**	**12 months**	**18 months**	**24 months**	**36 months**	
Kane J et al. [24].	28	Not specified		41%				0%
Crow T et al. [26].	120	Not specified				62%		46%
Gitlin M et al. [21].	53	3 months in remission		78%		96%		-
Wunderink L et al. [25].	161	6 months in remission			43%			21%
Chen E. et al. [22].	178	12 months +		79%				41%
Gaebel W. et al. [27].	44	12 months		57%				4%
Boonstra G. et al. [23].	20	12 months min remission	82%					12%
Emsley R et al. [10].	33	24 months		79%		94%	97%	-

It could be argued that a substantial proportion of patients remain relapse-free even 12 months after treatment discontinuation, and that these patients may not require continuous antipsychotic treatment. This may be an option if such patients could be identified prior to considering treatment discontinuation. However, while Chen et al. found pre-morbid schizoid and schizotypal traits, level of functioning, and a diagnosis of schizophrenia to significantly predict relapse after 12 months of treatment discontinuation, we were unable to identify any predictors of early relapse in our own study [10].

### Duration of antipsychotic treatment and risk of relapse after discontinuation

A key clinical question is what the risk of relapse is after a single episode of psychosis, and whether the risk is reduced with a longer treatment period. Despite the fact that several studies have assessed the risk of relapse after discontinuation of antipsychotic medication, there is no consensus on the recommended duration of treatment. A recent systematic review of guidelines for maintenance treatment of schizophrenia found that, of 14 such guidelines, discontinuation of antipsychotic medication after a first-episode of schizophrenia was partially recommended after 1 to 2 years in six, not recommended in two, and not mentioned in six [28].

It can be seen from Table 1 that the duration of treatment periods before withdrawal varied. There was no indication of reduced risk with longer treatment periods - in fact, the study with the longest prior treatment period (2 years) [10] had the highest risk of symptom recurrence. This has implications for clinical practice, and suggests that lengthening the recommended treatment period after a first psychotic episode does not reduce the risk of recurrence. It could even be speculated that a longer treatment duration might increase risk of relapse, although the study with the shortest reported treatment period (3 months in remission) had an equally high symptom recurrence rate [21].

### Time to relapse after treatment discontinuation

The interval between treatment discontinuation and symptom recurrence is highly variable. Three of the eight studies investigating relapse rates after treatment discontinuation (Table 1) reported mean times to relapse of 235 days [21], 323 days [27] and 207 days [10]. This could create the impression that patients remain well for a considerable period – perhaps months – before relapse. While this may be so for some, in many cases relapse occurs within days or weeks of discontinuation. In our own study all patients were treated with long-acting risperidone injection which is associated with the persistence of steady state blood levels for several weeks after the last injection [29]. Despite this several symptom recurrences occurred within a few weeks of the last injection, suggesting that relapse occurs with treatment reduction even in the presence of substantial antipsychotic occupancy of D2 receptors. Symptom recurrence shortly after treatment discontinuation is well recognized and has raised the question whether relapse may be a feature of drug withdrawal rather than the re-emergence of an underlying illness, at least in some patients [30]. The term “neuroleptic-induced supersensitivity psychosis” was originally proposed, and was linked to a hypothesised underlying mechanism of increased sensitivity of mesolimbic dopamine D2 receptors as a consequence of chronic D2 blockade with antipsychotic medication [31]. Moncrieff [30] suggested an alternative term of “rapid onset psychosis”, which does not imply underlying mechanisms. In a review article he pointed out that if the process of antipsychotic drug withdrawal is in itself psychotogenic, then the psychosis would be expected to have its own distinct symptom profile, different to that of the underlying illness. He underlined the need for further urgent research in this area. We were able to investigate this possibility by comparing the factor analysis derived symptom domains observed in the first psychotic episode with that of a recurrence episode. We found that symptom expression profiles were similar for the two episodes and, except for the positive domain, of similar severity [10]. While this counts against a role for a distinct antipsychotic withdrawal syndrome, we did not specifically examine the symptom profiles of those patients with rapid onset of relapse.

### Onset of relapse may be abrupt

Reliable early warning signs of relapse would be of great value in clinical settings. They would offer the opportunity of early intervention and prevention of florid relapse. Indeed, success has been reported in identifying early signs of relapse [32]. However, whereas the onset of a first episode of psychosis may be gradual and is typically heralded by a prodromal period lasting months to even years [33], this does not appear to be the case in relapse episodes. For these reasons it has been recommended that the term “prodromal symptoms” be restricted to precursors of a first psychotic episode and "early warning signs" be used to describe antecedents of psychotic relapse [34]. Studies suggest that it may be difficult to identify many patients who are at risk of imminent relapse in clinical practice, and that early warning signs are relatively unreliable predictors of relapse [35-37]. Using a structured interview for early signs of relapse, Herz and Mellville [38] assessed 145 patients with chronic schizophrenia and 80 family members. Approximately half of the patients and 68% of the family members indicated that the interval between the onset of noticeable symptoms and relapse was at least 1 week. Between 8 and 15% of patients and families gave this interval as 1 to 3 days and 7 to 8% patients and 11% families indicated that it was less than 1 day. Birchwood et al. [32] reported that 75% of relatives of patients noticed changes from 2 to 4 weeks before relapse. Henmi [39] retrospectively investigated 61 schizophrenic outpatients in remission, of whom 33 had relapses in the past 20 months. Non-specific (non-psychotic) prodromal symptoms were identified 4 weeks prior to relapse in 64%, although no changes were detected in global assessment of function. Tarrier et al. [40] compared symptoms of 16 patients who relapsed within 9 months after discharge with 16 patients who had not, and found a significant increase in depressive symptoms and hallucinations in the preceding month in those who had relapsed. In a review of the pertinent literature Norman and Malla [35] concluded that prodromal symptoms have only modest power as predictors of relapse. In a study investigating the predictive validity of self-reported early warning signs of relapse in a cohort of 60 remitted first-episode patients, the Early Signs Scale (ESS) achieved adequate sensitivity but poor specificity and positive predictive validity in predicting relapse or symptom exacerbations [41].

One difficulty in interpreting results of longitudinal studies assessing precursors of relapse is that the relapse events occur at different timepoints in different individuals. Therefore, group mean symptom scores at assessment visits do not give an accurate picture of symptom changes prior to relapse. To circumvent this problem we analysed precursors of relapse in a different way in two studies [10,11]. With the relapse visit as starting point we grouped patients with the preceding visits arranged before that (i.e. visit −1, -2, -3). We did not find a gradual worsening of symptoms prior to relapse, but rather that patients remained in remission right up until and including the visit prior to relapse. Retrospective estimates of caregivers indicated a mean duration 16.3±10.9 days between the onset of the first recognisable symptoms and psychotic symptom recurrence, with several patients relapsing within a matter of days [10]. Our analyses were conducted retrospectively and it is possible that with more frequent assessments and with the use of more sensitive instruments designed specifically to detect prodromal symptoms more subtle symptoms could have been detected. Nevertheless, the results suggest that in everyday clinical practice relying on early warning signs to prevent full-blown relapse would not be feasible. Frequent and careful follow-up together with the use of instruments specifically designed to detect early warning signs would be necessary [42].

### Once illness recurrence occurs symptom severity rapidly returns to levels similar to the initial psychotic episode

We observed a rapid return of symptoms in the relapse episode to severity levels similar to those in the acute phase of the first psychotic episode. This rapid return of symptoms occurred despite marked differences in estimated duration of untreated psychosis for the first-episode (129 ± 199 days) [43] and the second episode (16.3 ± 10.9 days) [12]. This finding is consistent with that of Chen et al. who found no significant correlation between symptom severity and time to receiving treatment [22].

### Is relapse associated with disease progression?

In addition to its associated psychosocial risks, active psychosis may affect the brain in a more fundamental way. It has been suggested that psychosis may be neurotoxic and that acute psychotic exacerbations represent active periods of a morbid process that leads to disease progression and to impairment of treatment response [4,44,45]. Supportive evidence was initially provided by the well replicated finding that a longer duration of untreated psychosis is associated with a poorer long-term outcome [46]. Similarly, relapses also represent episodes of active psychosis and may also be associated with illness progression. However, to date there is limited empirical evidence to support the neurotoxic psychosis hypothesis, and specifically for illness progression after relapse. A search of the literature revealed the following: 1) Treatment response has been observed to be better in first-episode schizophrenia than in chronic multi-episode schizophrenia [45]. 2) A study utilizing the neuroleptic threshold principle found that first-episode patients required lower doses of haloperidol to achieve optimal clinical response than multi-episode patients [47], raising the possibility that tolerance to the effects of antipsychotics may develop. 3) Eighty % of patients with schizophrenia were judged to have deteriorated over time in a 7-yr follow-up study, and the degree of deterioration was significantly correlated with the number of relapses that patients experienced [48]. 4) In another long-term follow-up study of the natural course of schizophrenia, Wiersma et al. examined the course of patients over the first four psychotic episodes. They reported a remarkable observation, namely that, on average 1 in 6 patients failed to remit after each episode, irrespective of which episode it was. 5) More direct evidence was obtained in a preliminary study comparing treatment response times of first and subsequent episodes, where median (SE) times to remission were significantly longer for the second episode and third episode [4]. On the other hand, there is evidence to suggest that disease progression does not occur as a consequence of relapse: 1) It has been reported that patients’ symptoms rapidly return to baseline with resumption of antipsychotic medication shortly after recurrence of psychotic symptoms [49]; 2) In patients with relatively refractory schizophrenia who were exposed to placebo treatment for at least 6 weeks and experienced symptom worsening, it was found that, given a sufficiently lengthy recovery period, symptom levels returned to those of baseline [50]; 3) Response trajectory analyses in early psychosis suggest that, while some patients respond poorly initially, response is generally characterised by amelioration [51]; 4) The clinical and neurobiological evidence for neurotoxicity of psychosis has been reviewed and found wanting [52]; 5. Finally, a recent paper challenges the concept of schizophrenia as a progressive brain disease, arguing the following: a) While longitudinal studies report a poor outcome in approximately 25% of patients, few of them describe incremental deterioration that would characterize neurodegenerative disorders; b) while some neuro-imaging studies report brain tissue volumes decreasing over time, this could be attributed to the effects of antipsychotic medication, substance abuse, effects of lifestyle or elevated glucocorticoid levels associated with chronic stress; c) cognitive functioning does not appear to deteriorate over time; and d) the deterioration that occurs in some patients could reflect poor access, or adherence, to treatment, the effects of concomitant conditions, and social and financial impoverishment [53].

We were able to compare treatment response before and after relapse in both a first-episode [12] and a multi-episode [11] sample. We found that in both groups treatment response was highly variable, with some patients rapidly returning to pre-relapse symptom severity levels, and others having a more protracted recovery. Most notably, we found evidence of emergent treatment failure in a subset of 16% of the first episode and 14% of the multi-episode samples respectively. These results are consistent with the findings of Wiersma et al. [54] who reported that on average 1 in 6 patients failed to recover from each of their first four relapses, irrespective of which relapse it was.

### Dopamine and the neurobiology of relapse in schizophrenia

In addition to the obvious clinical implications, our observations may shed light on the underlying pathogenesis of the illness. The very high relapse rates and abrupt re-emergence of psychotic symptoms suggest a reduced threshold for psychotic decompensation once a first episode has occurred [32], and are consistent with a direct relationship between dopamine and psychosis. The dopamine hypothesis of schizophrenia has been central to our understanding of neurobiological mechanisms underpinning the illness, and dopamine D2 receptor blockade remains a necessary and sufficient component for antipsychotic action [55]. In a recent elaboration of the dopamine hypothesis Howes and Kapur [56] incorporate multiple genetic and environmental factors that compromise the brain and manifest as dopamine dysregulation. They point out that elevated striatal dopamine function is likely linked to the dimension of psychosis rather than the full spectrum of symptom expression of the illness. Therefore relapse, characterised by acute psychotic exacerbation, would be associated with striatal dopamine hyperfunction, likely as elevation of presynaptic dopamine synthesis [57]. Linked to the dopamine hypothesis is the proposal that psychosis represents a state of aberrant salience due to a dysregulated hyperdopaminergic state [58]. A direct relationship is proposed between dopamine, psychosis and antipsychotic action, and supportive evidence is provided by the finding that the onset of antipsychotic effect is rapid rather than, as previously thought, delayed [59-61]. According to the aberrant salience hypothesis antipsychotics do not eradicate symptoms but dampen them. When they are discontinued there is a resurgence of a hyperdopaminergic state with symptoms being re-invested with salience [62], which would manifest as a rapid return of previous symptoms. This hypothesis is consistent with 1) the very high relapse rates reported after antipsychotic treatment discontinuation; 2) the failure of a longer treatment duration to reduce the risk of relapse when treatment is discontinued; 3) the onset of relapse soon after discontinuation in many patients; and 4) the abrupt onset of recurrence symptoms, with rapid return to levels of symptom severity that are similar to the previous episode.

The “glutamate” hypothesis of schizophrenia emerged from the observation that inhibition of glutamate N-methyl-D-aspartate (NMDA) receptors, with for example phencyclidine, induces an array of schizophrenia-likebehaviours in humans. Cortical and limbic striatal (nucleus accumbens) dopamine release is regulated by a glutamate-GABA-glutamate loop located on pyramidal cells of the frontal cortex [63]. Cortical hypoglutamatergia in turn compromises dopamine release in the ventral tegmentum leading to meso-limbic hyperdopaminergic and meso-cortical hypodopaminergia that we observe as positive or negative symptoms, respectively. Thus schizophrenia envelopes a combined state of glutamate *and* dopamine dysfunction [63]. However, altered glutamate may originate from various sources with activation of the NMDA receptor representing a distal event in the cascade, thereafter leading to the aforementioned dopamine changes. Schizophrenia is associated with a pro-inflammatory state [64], with altered blood/cerebrospinal fluid levels of interleukin-2 (IL-2), IL-6, IL-1, IL-10 and tumour necrosis factor (TNF)-α [65]. Mitochondrial dysfunction, altered pro-inflammatory cytokines and increased indoleamine 2,3 dioxygenase (IDO)-mediated conversion of tryptophan to quinolinic acid (an NMDA agonist) may directly or indirectly provoke glutamate hyperactivity leading to overt NMDA receptor activation, altered redox balance and oxidative stress. Importantly, each of the aforementioned either alone or in concert is capable of modifying dopamine release and function [66]. What is interesting is that increased CSF levels of IL-1 have been found to be a predictor of acute psychotic relapse in patients recently withdrawn from haloperidol [67]. Together this suggests that immune-inflammatory-glutamate dysfunction may play a critical role in the neurobiology of relapse. Indeed, the association between cytokine abnormalities and acute exacerbations of schizophrenia seems to be independent of antipsychotic medications [68]. In fact some cytokines (eg. IL-1β, IL-6, and TGF-β) as well as abnormal blood lymphocyte parameters (CD4/CD8) have been suggested to represent state markers for acute exacerbations of psychosis [69].

The pattern of treatment failure developing abruptly in a subset of patients suggests a threshold phenomenon, and is consistent with a sensitization, or “kindling” model. Such a model was initially proposed for TD where it was suggested that chronic antipsychotic exposure induces neostriatal dopaminergic receptor supersensitivity. Similarly, these same authors suggested that mesolimbic dopaminergic supersensitivity after chronic antipsychotic treatment could explain the emergence of antipsychotic treatment failure [31]. The kindling phenomenon has also been linked to increased excitatory glutamatergic activity combined with a relative loss of inhibitory GABA’ergic tone [70]. The vacuous chewing movement (VCM) model in rats has been proposed as a model for TD, and the involvement of dopamine receptor supersensitivity has been suggested [71]. Of note is that the VCM model is also associated with increased glutamate-nitric oxide signaling [72,73] and striatal oxidative stress [74] that has also been linked to antipsychotic discontinuation [75]. Antipsychotic-induced striatal oxidative stress is abolished with N-acetyl cysteine (NAC), a glutathione replenisher and NMDA receptor modulator [74]. Thus, dopamine receptor supersensitivity, glutamate dysfunction and the release of reactive oxygen and nitrogen species together may play a role in the development of TD. Drawing on this analogy with TD may be useful in explaining post-relapse emergence of treatment non-responsiveness. Indeed, clinical [76] and animal [66] studies concur that adjunctive NAC is an effective antipsychotic augmentation strategy. It could be postulated that a relative imbalance in GABA-glutamate function, altered redox balance and its associated effects on cellular resilience lead to a self-perpetuating morbid process that eventually compromises long-term treatment response and disease outcome. It is not clear whether intermittent treatment or chronic, uninterrupted treatment is more likely to induce supersensitivity. While some evidence suggests that interrupted antipsychotic treatment may increase the risk of supersensitivity [77], sensitization of D2 receptors and ensuing treatment failure has been reported in animals not only during antipsychotic withdrawal, but also as a “breakthrough” phenomenon during chronic antipsychotic administration [78].

## Conclusions

There is a need for further research on the phenomenology and neurobiology of relapse in schizophrenia. Particularly, the long-2222term outcome of patients with post-relapse treatment failure should be investigated to establish whether the emergent refractoriness persists. Given the difficulties in identifying those at risk of relapse, the ineffectiveness of rescue medications in preventing full-blown psychotic recurrence and the potentially serious consequences, every effort needs to be made to effectively address adherence and other factors predisposing to relapse. Antipsychotic treatment discontinuation should not be a part of routine clinical care and alternatives to the use of placebo in clinical trials need to be carefully reconsidered. However, despite the clearly demonstrated benefits of antipsychotic therapy in relapse prevention [79] it needs to be kept in mind that ongoing maintenance treatment carries its own burden, including substantial risk of relapse [11], significant side-effects and even the possibility of brain morphological changes [80].

## Competing interests

Robin Emsley has participated in speakers/advisory boards and received honoraria from AstraZeneca, Bristol-Myers Squibb, Janssen, Lilly, Lundbeck, Organon, Pfizer, Servier, Otsuka and Wyeth. He has received research funding from Janssen, Lundbeck and AstraZeneca. Bonginkosi Chiliza has participated in speakers/advisory boards and received honoraria from Janssen and Sandoz. Brian Harvey has participated in speakers/advisory boards and has received honoraria from Bristol-Myers Squibb, Organon (Merck), Pfizer and Servier, and has received research funding from Lundbeck. Laila Asmal has no conflict of interest to declare.

## Authors’ contributions

RE conceived and drafted the manuscript; BH was responsible for neurobiological inputs. BC and LA contributed to the conceptualisation and interpretation of results and drafting of the manuscript, and all authors reviewed and approved the final manuscript.

## Pre-publication history

The pre-publication history for this paper can be accessed here:

http://www.biomedcentral.com/1471-244X/13/50/prepub

## References

[B1] Robinson D Woerner MG Alvir JM Bilder R Goldman R Geisler S Predictors of relapse following response from a first episode of schizophrenia or schizoaffective disorder Arch Gen Psychiatry 1999 56 241 247 10.1001/archpsyc.56.3.241 10078501

[B2] Kane JM Treatment strategies to prevent relapse and encourage remission J Clin Psychiatry 2007 68 Suppl 14 27 30 18284275

[B3] Wyatt RJ Research in schizophrenia and the discontinuation of antipsychotic medications Schizophr Bull 1997 23 3 9 10.1093/schbul/23.1.3 9050108

[B4] Lieberman JA Alvir JM Koreen A Geisler S Chakos M Sheitman B Psychobiologic correlates of treatment response in schizophrenia Neuropsychopharmacology 1996 14 13S 21S 10.1016/0893-133X(95)00200-W 8866739

[B5] Alvarez-Jimenez M Priede A Hetrick SE Bendall S Killackey E Parker AG Risk factors for relapse following treatment for first episode psychosis: a systematic review and meta-analysis of longitudinal studies Schizophr Res 2012 139 116 128 10.1016/j.schres.2012.05.007 22658527

[B6] Coldham EL Addington J Addington D Medication adherence of individuals with a first episode of psychosis Acta Psychiatr Scand 2002 106 286 290 10.1034/j.1600-0447.2002.02437.x 12225495

[B7] Tiihonen J Haukka J Taylor M Haddad PM Patel MX Korhonen P A nationwide cohort study of oral and depot antipsychotics after first hospitalization for schizophrenia Am J Psychiatry 2011 168 603 609 10.1176/appi.ajp.2011.10081224 21362741

[B8] Miller BJ A review of second-generation antipsychotic discontinuation in first-episode psychosis J Psychiatr Pract 2008 14 289 300 10.1097/01.pra.0000336756.65308.83 18832960

[B9] Miller BJ Bodenheimer C Crittenden K Second-Generation Antipsychotic Discontinuation in First Episode Psychosis: An Updated Review Clin Psychopharmacol Neurosci 2011 9 45 53 23429653 10.9758/cpn.2011.9.2.45 PMC3569083

[B10] Emsley R Oosthuizen PP Koen L Niehaus DJ Martinez L Symptom Recurrence Following Intermittent Treatment in First Episode Schizophrenia Successfully Treated for Two Years In J Clin Psychiatry 2012 In press 10.4088/JCP.11m07138 22579160

[B11] Emsley R Nuamah I Hough D Gopal S Treatment response after relapse in a placebo controlled maintenance trial in schizophrenia Schizophr Res 2012 138 1 29 34 Epub 2012 Mar 23 10.1016/j.schres.2012.02.030 22446143

[B12] Emsley R Oosthuizen PP Koen L Niehaus DJ Martinez L Comparison of Treatment Response in Second Episode Versus First Episode Schizophrenia J Clin Psychopharmaco 2013 33 1 80 83 10.1097/JCP.0b013e31827bfcc1 23277247

[B13] Moller HJ von Ziersen D Course and outcome of schizophrenia Schizophrenia 1995 Oxford: Blackwell 127

[B14] Kane JM Jeste DV Barnes TR Tardive dyskinesia: A task force report of the American Psychiatric Association 1992 Washington, D.C: American Psychiatric Association Ref Type: Report

[B15] Meltzer HY Neuroleptic withdrawal in schizophrenic patients An idea whose time has come. Arch Gen Psychiatry 1995 52 200 202 10.1001/archpsyc.1995.03950150032005 7872845

[B16] Newcomer JW Metabolic considerations in the use of antipsychotic medications: a review of recent evidence J Clin Psychiatry 2007 68 Suppl 1 20 27 17286524

[B17] Bostwick JR Guthrie SK Ellingrod VL Antipsychotic-induced hyperprolactinemia Pharmacotherapy 2009 29 64 73 10.1592/phco.29.1.64 19113797

[B18] Correll CU Schenk EM Tardive dyskinesia and new antipsychotics Curr Opin Psychiatry 2008 21 151 156 10.1097/YCO.0b013e3282f53132 18332662

[B19] Perkins DO Gu H Weiden PJ McEvoy JP Hamer RM Lieberman JA Predictors of treatment discontinuation and medication nonadherence in patients recovering from a first episode of schizophrenia, schizophreniform disorder, or schizoaffective disorder: a randomized, double-blind, flexible-dose, multicenter study J Clin Psychiatry 2008 69 106 113 10.4088/JCP.v69n0114 18312044

[B20] Gilbert PL Harris MJ McAdams LA Jeste DV Neuroleptic withdrawal in schizophrenic patients. A review of the literature. Arch Gen Psychiatry 1995 52 173 188 10.1001/archpsyc.1995.03950150005001 7872841

[B21] Gitlin M Nuechterlein K Subotnik KL Ventura J Mintz J Fogelson DL Clinical outcome following neuroleptic discontinuation in patients with remitted recent-onset schizophrenia Am J Psychiatry 2001 158 1835 1842 10.1176/appi.ajp.158.11.1835 11691689

[B22] Chen EY Hui CL Lam MM Chiu CP Law CW Chung DW Maintenance treatment with quetiapine versus discontinuation after one year of treatment in patients with remitted first episode psychosis: randomised controlled trial BMJ 2010 341 c4024 10.1136/bmj.c4024 20724402 PMC2924475

[B23] Boonstra G Burger H Grobbee DE Kahn RS Antipsychotic prophylaxis is needed after remission from a first psychotic episode in schizophrenia patients: results from an aborted randomised trial Int J Psychiatry Clin Pract 2011 15 128 134 10.3109/13651501.2010.534801 22121861

[B24] Kane JM Rifkin A Quitkin F Nayak D Ramos-Lorenzi J Fluphenazine vs placebo in patients with remitted, acute first-episode schizophrenia Arch Gen Psychiatry 1982 39 70 73 10.1001/archpsyc.1982.04290010048009 6275811

[B25] Wunderink L Nienhuis FJ Sytema S Slooff CJ Knegtering R Wiersma D Guided discontinuation versus maintenance treatment in remitted first-episode psychosis: relapse rates and functional outcome J Clin Psychiatry 2007 68 654 661 10.4088/JCP.v68n0502 17503973

[B26] Crow TJ MacMillan JF Johnson AL Johnstone EC A randomised controlled trial of prophylactic neuroleptic treatment In Br J Psychiatry 1986 148 120 127 10.1192/bjp.148.2.120 2870753

[B27] Gaebel W Riesbeck M Wolwer W Klimke A Eickhoff M Von WM Relapse prevention in first-episode schizophrenia–maintenance vs intermittent drug treatment with prodrome-based early intervention: results of a randomized controlled trial within the German Research Network on Schizophrenia J Clin Psychiatry 2011 72 205 218 10.4088/JCP.09m05459yel 20673559

[B28] Takeuchi H Suzuki T Uchida H Watanabe K Mimura M Antipsychotic treatment for schizophrenia in the maintenance phase: a systematic review of the guidelines and algorithms Schizophr Res 2012 134 219 225 10.1016/j.schres.2011.11.021 22154594

[B29] Gefvert O Eriksson B Persson P Helldin L Bjorner A Mannaert E Pharmacokinetics and D2 receptor occupancy of long-acting injectable risperidone (Risperdal Consta) in patients with schizophrenia Int J Neuropsychopharmacol 2005 8 27 36 10.1017/S1461145704004924 15710053

[B30] Moncrieff J Does antipsychotic withdrawal provoke psychosis? Review of the literature on rapid onset psychosis (supersensitivity psychosis) and withdrawal-related relapse Acta Psychiatr Scand 2006 114 3 13 16774655 10.1111/j.1600-0447.2006.00787.x

[B31] Chouinard G Jones BD Neuroleptic-induced supersensitivity psychosis: clinical and pharmacologic characteristics Am J Psychiatry 1980 137 16 21 6101522 10.1176/ajp.137.1.16

[B32] Birchwood M Smith J MacMillan F Hogg B Prasad R Harvey C Predicting relapse in schizophrenia: the development and implementation of an early signs monitoring system using patients and families as observers, a preliminary investigation Psychol Med 1989 19 649 656 10.1017/S0033291700024247 2798634

[B33] Yung AR McGorry PD The prodromal phase of first-episode psychosis: past and current conceptualizations Schizophr Bull 1996 22 353 370 10.1093/schbul/22.2.353 8782291

[B34] Bustillo J Buchanan RW Carpenter WT Jr: Prodromal symptoms vs. early warning signs and clinical action in schizophrenia. Schizophr Bull 1995 21 553 559 10.1093/schbul/21.4.553 8749883

[B35] Norman RM Malla AK Prodromal symptoms of relapse in schizophrenia: a review Schizophr Bull 1995 21 527 539 10.1093/schbul/21.4.527 8749881

[B36] Gaebel W Riesbeck M Revisiting the relapse predictive validity of prodromal symptoms in schizophrenia Schizophr Res 2007 95 19 29 10.1016/j.schres.2007.06.016 17630253

[B37] Gaebel W Frick U Kopcke W Linden M Muller P Muller-Spahn F Early neuroleptic intervention in schizophrenia: are prodromal symptoms valid predictors of relapse? Br J Psychiatry Suppl 1993 21 8 12 8105814

[B38] Herz MI Melville C Relapse in schizophrenia Am J Psychiatry 1980 137 801 805 6104444 10.1176/ajp.137.7.801

[B39] Henmi Y Prodromal symptoms of relapse in schizophrenic outpatients: retrospective and prospective study Jpn J Psychiatry Neurol 1993 47 753 775 7911165 10.1111/j.1440-1819.1993.tb01824.x

[B40] Tarrier N Barrowclough C Bamrah JS Prodromal signs of relapse in schizophrenia Soc Psychiatry Psychiatr Epidemiol 1991 26 157 161 1948295 10.1007/BF00795207

[B41] Gleeson JF Rawlings D Jackson HJ McGorry PD Early warning signs of relapse following a first episode of psychosis Schizophr Res 2005 80 107 111 10.1016/j.schres.2005.07.019 16125373

[B42] Birchwood M Spencer E Early intervention in psychotic relapse Clin Psychol Rev 2001 21 1211 1226 10.1016/S0272-7358(01)00105-2 11702513

[B43] Emsley R Medori R Koen L Oosthuizen PP Niehaus DJ Rabinowitz J Long-acting injectable risperidone in the treatment of subjects with recent-onset psychosis: a preliminary study J Clin Psychopharmacol 2008 28 210 213 10.1097/JCP.0b013e318167269d 18344732

[B44] Wyatt RJ Neuroleptics and the natural course of schizophrenia Schizophr Bull 1991 17 325 351 10.1093/schbul/17.2.325 1679255

[B45] Lieberman JA Jody D Geisler S Alvir J Loebel A Szymanski S Time course and biologic correlates of treatment response in first-episode schizophrenia Arch Gen Psychiatry 1993 50 369 376 10.1001/archpsyc.1993.01820170047006 8098203

[B46] Marshall M Lewis S Lockwood A Drake R Jones P Croudace T Association between duration of untreated psychosis and outcome in cohorts of first-episode patients: a systematic review Arch Gen Psychiatry 2005 62 975 983 10.1001/archpsyc.62.9.975 16143729

[B47] McEvoy JP Hogarty GE Steingard S Optimal dose of neuroleptic in acute schizophrenia A controlled study of the neuroleptic threshold and higher haloperidol dose. Arch Gen Psychiatry 1991 48 739 745 10.1001/archpsyc.1991.01810320063009 1883257

[B48] Curson DA Barnes TR Bamber RW Platt SD Hirsch SR Duffy JC Long-term depot maintenance of chronic schizophrenic out-patients: the seven year follow-up of the Medical Research Council fluphenazine/placebo trial. III. Relapse postponement or relapse prevention? The implications for long-term outcome Br J Psychiatry 1985 146 474 480 10.1192/bjp.146.5.474 3893600

[B49] Glovinsky D Kirch DG Wyatt RJ Early antipsychotic response to resumption of neuroleptics in drug-free chronic schizophrenic patients Biol Psychiatry 1992 31 968 970 10.1016/0006-3223(92)90124-I 1637936

[B50] Wyatt RJ Henter ID Bartko JJ The long-term effects of placebo in patients with chronic schizophrenia Biol Psychiatry 1999 46 1092 1105 10.1016/S0006-3223(99)00227-9 10536745 PMC4303345

[B51] Levine SZ Leucht S Elaboration on the early-onset hypothesis of antipsychotic drug action: treatment response trajectories Biol Psychiatry 2010 68 86 92 10.1016/j.biopsych.2010.01.012 20227681

[B52] McGlashan TH Is active psychosis neurotoxic? Schizophr Bull 2006 32 609 613 16914639 10.1093/schbul/sbl032 PMC2632265

[B53] Zipursky RB Reilly JL Murray RM The myth of schizophrenia as a progressive brain disease Schizophr Bull 2012 Epub ahead of print 10.1093/schbul/sbs135 PMC3796078 23172002

[B54] Wiersma D Nienhuis FJ Slooff CJ Giel R Natural course of schizophrenic disorders: a 15-year followup of a Dutch incidence cohort Schizophr Bull 1998 24 75 85 10.1093/oxfordjournals.schbul.a033315 9502547

[B55] Kapur S Mamo D Half a century of antipsychotics and still a central role for dopamine D2 receptors Prog Neuropsychopharmacol Biol Psychiatry 2003 27 1081 1090 10.1016/j.pnpbp.2003.09.004 14642968

[B56] Howes OD Kapur S The dopamine hypothesis of schizophrenia: version III–the final common pathway Schizophr Bull 2009 35 549 562 10.1093/schbul/sbp006 19325164 PMC2669582

[B57] Howes OD Kambeitz J Kim E Stahl D Slifstein M Abi-Dargham A The Nature of Dopamine Dysfunction in Schizophrenia and What This Means for Treatment: Meta-analysis of Imaging Studies Arch Gen Psychiatry 2012 69 8 776 786 10.1001/archgenpsychiatry.2012.169 22474070 PMC3730746

[B58] Kapur S Psychosis as a state of aberrant salience: a framework linking biology, phenomenology, and pharmacology in schizophrenia Am J Psychiatry 2003 160 13 23 10.1176/appi.ajp.160.1.13 12505794

[B59] Agid O Kapur S Arenovich T Zipursky RB Delayed-onset hypothesis of antipsychotic action: a hypothesis tested and rejected Arch Gen Psychiatry 2003 60 1228 1235 10.1001/archpsyc.60.12.1228 14662555

[B60] Leucht S Busch R Hamann J Kissling W Kane JM Early-onset hypothesis of antipsychotic drug action: a hypothesis tested, confirmed and extended Biol Psychiatry 2005 57 1543 1549 10.1016/j.biopsych.2005.02.023 15953491

[B61] Emsley R Rabinowitz J Medori R Time course for antipsychotic treatment response in first-episode schizophrenia Am J Psychiatry 2006 163 743 745 10.1176/appi.ajp.163.4.743 16585455

[B62] Kapur S Agid O Mizrahi R Li M How antipsychotics work-from receptors to reality NeuroRx 2006 3 10 21 10.1016/j.nurx.2005.12.003 16490410 PMC3593357

[B63] Schwartz TL Sachdeva S Stahl SM Glutamate neurocircuitry: theoretical underpinnings in schizophrenia Front Pharmacol 2012 3 195 23189055 10.3389/fphar.2012.00195 PMC3505861

[B64] Leonard BE Schwarz M Myint AM The metabolic syndrome in schizophrenia: is inflammation a contributing cause? J Psychopharmacol 2012 26 33 41 10.1177/0269881111431622 22472311

[B65] Drzyzga L Obuchowicz E Marcinowska A Herman ZS Cytokines in schizophrenia and the effects of antipsychotic drugs Brain Behav Immun 2006 20 532 545 10.1016/j.bbi.2006.02.002 16580814

[B66] Moller M Du Preez JL Viljoen FP Berk M Emsley R Harvey BH Social isolation rearing induces mitochondrial, immunological, neurochemical and behavioural deficits in rats, and is reversed by clozapine or N-acetyl cysteine Brain Behavior and Immunity Epub ahead of print 10.1016/j.bbi.2012.12.011 23270677

[B67] McAllister CG van Kammen DP Rehn TJ Miller AL Gurklis J Kelley ME Increases in CSF levels of interleukin-2 in schizophrenia: effects of recurrence of psychosis and medication status Am J Psychiatry 1995 152 1291 1297 7653683 10.1176/ajp.152.9.1291

[B68] Miller BJ Buckley P Seabolt W Mellor A Kirkpatrick B Meta-analysis of cytokine alterations in schizophrenia: clinical status and antipsychotic effects Biol Psychiatry 2011 70 663 671 10.1016/j.biopsych.2011.04.013 21641581 PMC4071300

[B69] Miller BJ Gassama B Sebastian D Buckley P Mellor A Meta-Analysis of Lymphocytes in Schizophrenia: Clinical Status and Antipsychotic Effects Biol Psychiatry 2012 doi:pii: S0006-3223(12):00779-2.doi:10.1016/j.biopsych.2012.09.007 10.1016/j.biopsych.2012.09.007 PMC3816144 23062357

[B70] Harvey BH McEwen BS Stein DJ Neurobiology of antidepressant withdrawal: implications for the longitudinal outcome of depression Biol Psychiatry 2003 54 1105 1117 10.1016/S0006-3223(03)00528-6 14625154

[B71] Andreassen OA Jorgensen HA Neurotoxicity associated with neuroleptic-induced oral dyskinesias in rats, implications for tardive dyskinesia Prog Neurobiol 2000 61 525 531 10.1016/S0301-0082(99)00064-7 10748322

[B72] Naidu PS Kulkarni SK Excitatory mechanisms in neuroleptic-induced vacuous chewing movements (VCMs): possible involvement of calcium and nitric oxide Behav Pharmacol 2001 12 209 216 10.1097/00008877-200105000-00006 11485057

[B73] Nel A Harvey BH Haloperidol-induced dyskinesia is associated with striatal NO synthase suppression: reversal with olanzapine Behav Pharmacol 2003 14 251 255 10.1097/00008877-200305000-00010 12799528

[B74] Harvey BH Joubert C du Preez JL Berk M Effect of chronic N-acetyl cysteine administration on oxidative status in the presence and absence of induced oxidative stress in rat striatum Neurochem Res 2008 33 508 517 10.1007/s11064-007-9466-y 17763945

[B75] Harvey BH Bester A Withdrawal-associated changes in peripheral nitrogen oxides and striatal cyclic GMP after chronic haloperidol treatment Behav Brain Res 2000 111 203 211 10.1016/S0166-4328(00)00156-X 10840145

[B76] Berk M Copolov D Dean O Lu K Jeavons S Schapkaitz I N-acetyl cysteine as a glutathione precursor for schizophrenia–a double-blind, randomized, placebo-controlled trial Biol Psychiatry 2008 64 361 368 10.1016/j.biopsych.2008.03.004 18436195

[B77] van Harten PN Hoek HW Matroos GE Koeter M Kahn RS Intermittent neuroleptic treatment and risk for tardive dyskinesia: Curacao Extrapyramidal Syndromes Study III Am J Psychiatry 1998 155 565 567 9546009 10.1176/ajp.155.4.565

[B78] Samaha AN Seeman P Stewart J Rajabi H Kapur S "Breakthrough" dopamine supersensitivity during ongoing antipsychotic treatment leads to treatment failure over time J Neurosci 2007 27 2979 2986 10.1523/JNEUROSCI.5416-06.2007 17360921 PMC6672560

[B79] Leucht S Tardy M Komossa K Heres S Kissling W Salanti G Antipsychotic drugs versus placebo for relapse prevention in schizophrenia: a systematic review and meta-analysis Lancet 2012 379 2063 2071 10.1016/S0140-6736(12)60239-6 22560607

[B80] Konopaske GT Dorph-Petersen KA Sweet RA Pierri JN Zhang W Sampson AR Effect of chronic antipsychotic exposure on astrocyte and oligodendrocyte numbers in macaque monkeys Biol Psychiatry 2008 63 759 765 10.1016/j.biopsych.2007.08.018 17945195 PMC2386415

